# Genome-wide identification and characterization of *SnRK* family genes in *Brassica napus*

**DOI:** 10.1186/s12870-020-02484-3

**Published:** 2020-06-22

**Authors:** Weizhuo Zhu, Dezhi Wu, Lixi Jiang, Lingzhen Ye

**Affiliations:** Institute of Crop Science, Zhejiang University, Hangzhou, 310058 China

**Keywords:** *Brassica napus*, *SnRK*, Genome-wide, Abiotic stress, Expression patterns

## Abstract

**Background:**

Sucrose non-fermenting 1 related protein kinases (*SnRK)* play crucial roles in responding to biotic and abiotic stresses through activating protein phosphorylation pathways. However, little information of *SnRK* genes was available in *Brassica napus*, one of important oil crops. Recently, the released sequences of the reference genome of *B.napus* provide a good chance to perform genome-wide identification and characterization of *BnSnRK* gene family in the rapeseed.

**Results:**

Totally 114 *SnRK* genes distributed on 19 chromosomes were identified in the genome of *B.napus* and classified into three subfamilies on the basis of phylogenetic analysis and the domain types. According to gene structure and motif composition analysis, the *BnSnRK* sequences showed obvious divergence among three subfamilies. Gene duplication and synteny between the genomes of the rapeseed and *Arabidopsis* were also analyzed to provide insights into the evolutionary characteristics of *BnSnRK* family genes. C*is-*element analysis revealed that *BnSnRKs* may response to diverse environmental stresses. Moreover, the expression patterns of *BnSnRKs* in various tissues and under diverse abiotic stresses were distinct difference. Besides, Single Nucleotide Polymorphisms (SNP) distribution analysis suggests the function disparity of *BnSnRK* family genes in different genotypes of the rapeseed.

**Conclusion:**

We examined genomic structures, evolution features, expression patterns and SNP distribution of 114 *BnSnRKs*. The results provide valuable information for functional characterization of *BnSnRK* genes in future studies.

## Background

Plants develop various molecular defense mechanisms to cope with abiotic stresses, including salinity, drought and cold stresses. Gene expression regulation and protein modification are two important ways for plants to deal with these stresses [1]. The processes of phosphorylation and dephosphorylation mediated by protein kinase play crucial roles in protein modification [2]. Among the reported protein kinase genes, sucrose non-fermenting 1 (SNF1)-related protein kinases (*SnRKs*) are involved in different physiological processes [3].

In plants, *SnRKs* could be divided into three subfamilies: *SnRK1*, *SnRK2* and *SnRK3* based on their sequence similarity and gene structures [3, 4]. In detail, the *SnRK1* subfamily contain a highly conserved N-terminal protein kinase (Pkinase) domain, which are the homologous genes of *SNF1* in yeasts and *AMPKs* in mammals [5]. The other subfamilies *SnRK2/3* are unique in plants, both are more diverse in plants than the *SnRK1* subfamily members. The member of *SnRK2* harbors a conserved Pkinase domain and a C-terminal variable adjusting conserved domain [6]. In addition, *SnRK3* known as *CIPK* (CBL-interacting protein kinases), also contain conserved N-terminal protein kinase domains and NAF domains, PPI domains in C-terminal [7, 8].

The *SnRK1* family genes involve in the response of plant cells to starvation and metabolic stress. *SnRK1* kinases are the catalytic subunits of heterotrimeric complexes that interact with two other subunits [9]. In *Solanum tuberosum, SnRK1* was proved to be involved in induction of sucrose synthase expression and played an important role in carbohydrate metabolism regulation [10]. Besides, low energy stress (e.g. darkness and hypoxia) could trigger *SnRK1α* nuclear translocation and further induce *SnRK1* target genes to refresh cellular energy for plant growth [11]. Furthermore, considerable evidences indicated that *SnRK1* genes were hubs in a network of various signaling pathways including cell cycle regulation, pathogen responses and meristem development [12].

On the other hand, the *SnRK2* genes play important roles in responding to abiotic stresses in plants, especially for osmotic and salt stress. For instance, *SnRK2.10* phosphorylate several target genes including dehydrins *ERD10* and *ERD14* to deal with osmotic stress in *Arabidopsis thaliana* [13]. *SnRK2.1* positivly regulate salt tolerance in *Nicotiana tabacum* [14, 15]. In *A. thaliana*, *SnRK2* subfamily genes could be classified into three groups: group 1 contains the ABA-independent kinases, group 2 includes genes responding to drought stress, and group 3 kinases are strongly stimulated by ABA [6, 16]. The current researches on ABA-dependent group 3 kinases are the most extensive. For example, *AtSnRK2*.6 (OST1), one of the *SnRK2 family gene,* plays a core role in the ABA signaling pathway in stomatal guard cells, and *OST1* protein stability can be regulated via E3-ubiquitin ligase *HOS15* to reduce ABA signal sensitivity in *Arabidopsis* [17, 18].

*SnRK3* kinases known as *CIPKs* (CBL (calcium sensor calcineurin B-like proteins)-interacting protein kinases), perform vital functions in resistance to various stresses in plants [3, 19]. For example, *SOS* (salt overly sensitive) system was the first discoverd *CBL-CIPK* pathway in *A.thaliana*. In detail, the calcium signal produced by salt stress was sensed by SOS3 (*AtCBL4*) on cell membrane, and then SOS3 combined with *SOS2* (*AtCIPK24*) forming complex to phosphorylate SOS1 (Na^+^/H^+^ antiporter) to remove excess Na^+^ out of root cells [20, 21]. Besides, *MdCIPK13* and *MdCIPK22* enhanced salt and drought tolerance through targeting sucrose transporter *MdSUT2.2* for phosphorylation in apple [22, 23]. Overexpression of *BnCBL1-BnCIPK6* in *B.napus* could enhance high salinity tolerance and low potassium tolerance [24]. In conclusion, increasing evidences emphasized the importance of *SnRKs* function in nutrition utilization and stress response, and finally researchers could improve plants resistance to stresses by genetic manipulation using these genes.

*Brassica napus* is an important oil crop in the world. Recently, the genomes of Darmor-*bzh* (winter ecotype), Zhongshuang 11 and NY7 (seni-winter ecotype) were successfully sequenced and assembled [25–27]; however, the systematic analysis of *BnSnRK* gene family has been not well reported. In this study, 114 *SnRK* gene members were identified in the *B.napus* genome. We systematically analyzed their phylogenetic relationships, protein motifs, gene structures, chromosome distributions and *cis-*elements in promoter regions. Moreover, the expression profiles of *BnSnRKs* in diverse tissues as well as under abiotic stresses were determined. In addition, SNPs of each *BnSnRK* gene were systematically identified in a worldwide collection with 300 core germplasm accessions. These results will provide useful information for further investigation of molecular mechanisms of *BnSnRK* genes for abiotic stress tolerance and molecular breeding in *B.napus*.

## Results

### Identification and phylogenetic analysis of *SnRK* genes in *B.napus*

A total of 114 proteins with Ser/Thr kinase domain were identified as the members of SnRK family in the *B.napus* genome (Table S2). The longest amino acid sequence of each protein was selected for further analyses. The information of gene names, ID, chromosomal locations, amino acid numbers, molecular weights (MW), isoelectric points (pI) and domains were listed in Tables S1 and S2. The amino acid length of 114 *BnSnRKs* protein is ranged from 190 to 1241, correspondingly the melocular weight ranges from 22.0 to 140.6 kDa. The coding regions and sequences of each gene were listed in Table S3.

**Fig. 1:**
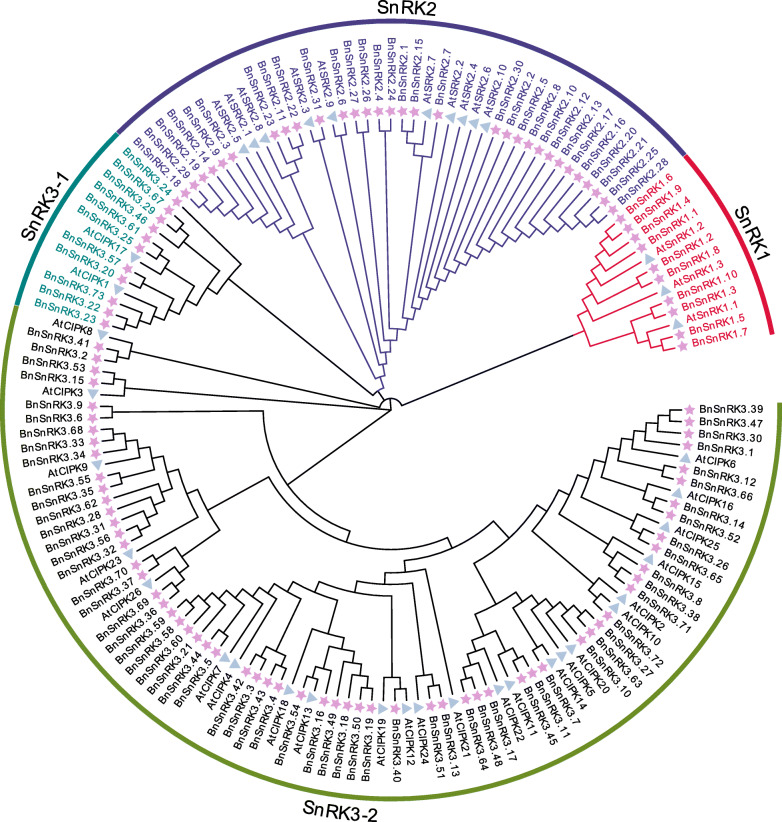
Phylogenetic tree of *SnRK* families in *B.napus* and *A.thaliana*. The different-colored arcs indicate different groups (or subgroups) of the *SnRKs*. The unrooted Neighbour-Joining phylogenetic tree was constructed using MEGA7 with full-length amino acid sequences of 153 *SnRKs*, and the bootstrap test replicate was set as 1000 times. The five-point stars and triangles represent the *SnRK* proteins from *B. napus* and *A. thaliana*, respectively

To analyze evolutionary relationships of *SnRK* genes in *B.napus* and *A.thaliana*, an unrooted phylogenetic tree was constructed using the full-length amino acid sequences of all *SnRKs*. Totally, 39 *SnRKs* from *A.thaliana* and 114 *SnRKs* from *B.napus* were identified and used in the study (Fig. 1). It was reported that 39 *AtSnRKs* could be clustered into three groups [3]. In this study, based on the phylogenetic analysis, 114 *BnSnRKs* were also classfied into three groups. In details, 10 proteins are in *BnSnRK1* subfamily with Pkinase (PF00069 of Pfam), UBA (PF00627) and KA1 (PF02149) domains, excepting *BnSnRK1.3*, while 31 proteins contain pkinase domain in *BnSnRK2* subfamily with high similarity to *AtSnRK2* subfamily, and 73 proteins with Pkinase and NAF (PF03822) domains are in *SnRK3* subfamily, respectively (Fig. 1).

### Gene structure and protein motif analysis of *BnSnRK* genes

**Fig. 2:**
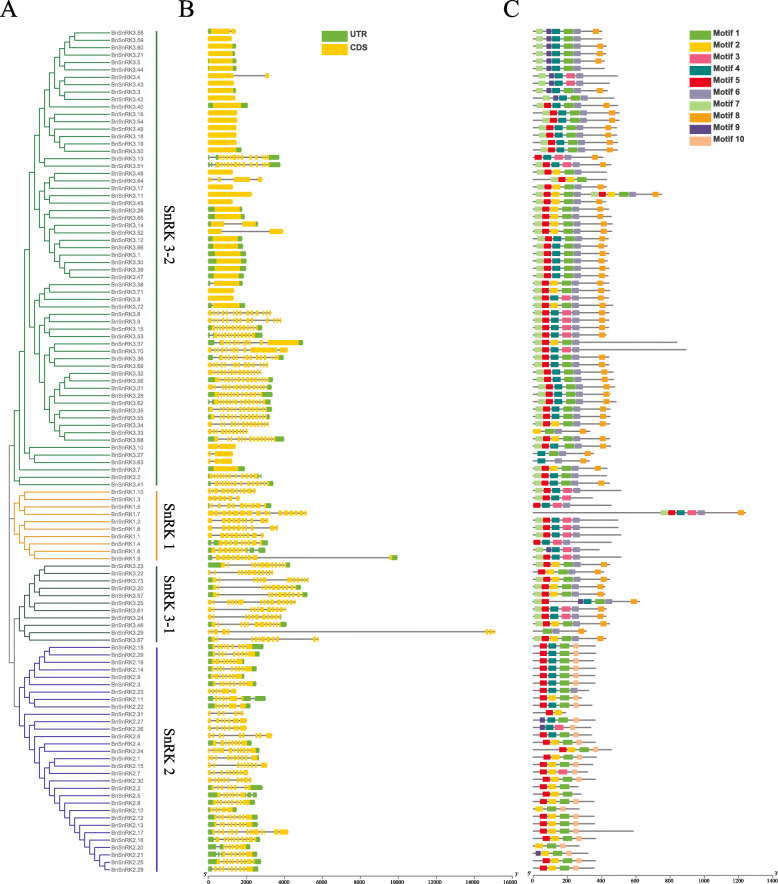
Phylogenetic relationships, architecture of conserved protein motifs and gene structure of the *SnRK* genes from *B.napus*. **a** Phylogenetic tree of 104 *BnSnRK* proteins. The unrooted neighbor-joining phylogenetic tree was constructed with MEGA7 using full-length amino acid sequences of 104 *BnSnRK* proteins, and the bootstrap test replicate was set as 1000 times. **b** Exon/intron organization of *BnSnRK* genes. Yellow boxes represent exons and black lines with same length represent introns. The upstream/downstream region of *BnSnRK* genes are indicated in green boxes. The length of exons can be inferred by the scale at the bottom. **c** The motif composition of *BnSnRK* proteins. The motifs, numbers 1–10, are displayed in different colored boxes. The sequence information for each motif is provided in Table S4. The length of protein can be estimated using the scale at the bottom

The web server GSDS (Gene Structure Display Server) analysis was done to determine gene structures of *BnSnRK*s. As shown in Fig. 2, one to fifteen exons were found in these *BnSnRK* genes. The *BnSnRK1* subfamily genes have more than 6 exons, and 6 to 13 exons for the Bn*SnRK2* subfamily genes. While it is various for the *BnSnRK3* subfamily with ranged from 1 to 15 exons. Furthermore, there are two subgroups in the *BnSnRK3* family. The genes in the *SnRK3* subgroup 1 contained more than 8 exons, while the genes in the subgroup 2 contain less than 4 exons.

The schematic structures of all *BnSnRK* proteins were constructed using the MEME (Multiple Em for Motif Elicitation) motif analysis results (Fig. 2c). The sequence and length information of the conserved motif were shown in Supplementary Table S4. In this study, it was found that all *BnSnRK* genes retained the conserved Pkinase domain containing the motif 1, 2, 3, 4, 5 (Fig. 2c). Moreover, *BnSnRK* genes within the same subfamily share a similar motif composition, while *BnSnRK* genes between diverse subfamilies showed distinct differences in motif composition. In detail, *BnSnRK1* subfamily genes contain motif 3, 4, 5, 6, 7; *BnSnRK2* genes have motif 1, 2, 5, 6, 10 or motif 1, 4, 5, 6, 10; *BnSnRK3–1* genes contain motif 1, 2, 5, 6, 7, 8 or motif 1, 4, 5, 6, 7, 8; *BnSnRK3–2* genes have motif 1, 5, 6, 7, 8 or motif 1, 6, 7, 8, 9 (Fig. 2c). In summary, the conserved motif compositions and similar gene structures of the *SnRK* genes within the same subfamily, strongly support the reliability of the subfamily classifications by phylogenetic analysis.

### Chromosomal distribution, genome synteny and gene duplication of *BnSnRK* genes

**Fig. 3:**
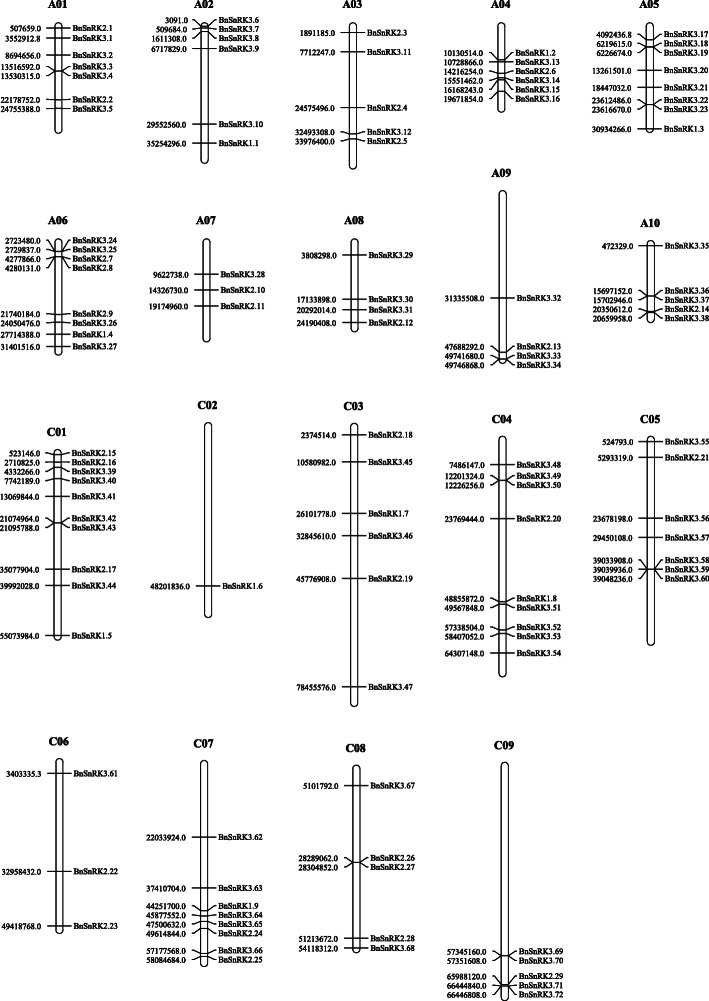
Chromosomal distribution of *SnRK* genes in *Brassica napus*. Left number represent physical location on chromosomes of *BnSnRKs*

Chromosomal analysis showed that 110 *BnSnRK* genes were distributed over 19 chromosomes, excepting 4 genes (*BnSnRK1.10, BnSnRK2.30, BnSnRK2.31, BnSnRK3.73)* could not be mapped into chromosomes of the rapeseed genome (Fig. 3). Among them, 56 *BnSnRK* genes were located in the AA subgenome, including 4 *BnSnRK1* genes, 14 *BnSnRK2* genes, 38 *BnSnRK3* genes; while 54 genes were located in the CC subgenome, containing 5 *BnSnRK1*genes, 15 *BnSnRK2* genes, 34 *BnSnRK3* genes. In addition, some *BnSnRK2* and *BnSnRK3* subfamily genes were formed as clusters in diverse chromosomes, such as *BnSnRK3.22* and *3.23* (Fig. 3). These results indicated that *BnSnRK* genes were randomly distributed in the chromosomes of the AA or CC subgenome.

**Fig. 4:**
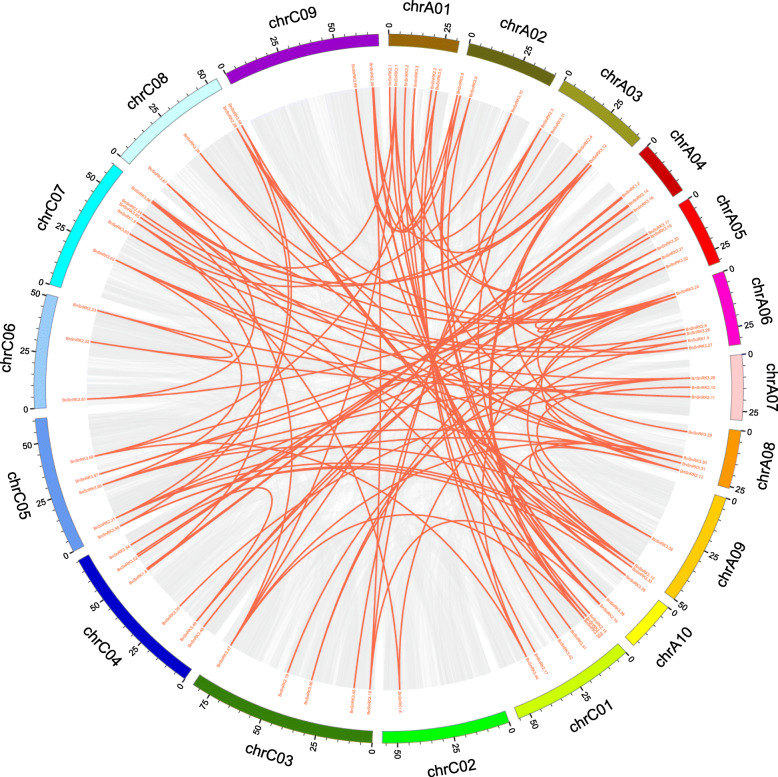
the synteny analysis of *BnSnRK* family in *B.napus*. Gray lines indicate all synteny blocks in the *B.napus* genome, and the red lines indicate duplicated *BnSnRK* gene pairs. The chromosome number is indicated at the bottom of each chromosome

Using BLAST and MCScanX methods, 106 segmental duplication events were identified (Fig. 4 and Table S5). Among these events, 104 events were happened between diverse chromosomes, while only 2 duplication events were detected within same chromosome. The result suggested that part of *BnSnRK* genes were possibly generated by gene duplication, and the segmental duplication events played vital roles in the expansion of *SnRK* genes in the *B.napus* genome. We also analyzed the occurrence of the tandem duplication events. Here, 25 tightly linked *BnSnRK* genes (*BnSnRK2*.7/2.8, *BnSnRK3*.3/3.4, *BnSnRK3*.18/3.19, *BnSnRK3*.22/3.23, *BnSnRK3*.24/3.25, *BnSnRK3*.33/3.34, *BnSnRK3*.36/3.37, *BnSnRK3*.42/3.43, *BnSnRK3*.49/3.50, *BnSnRK3*.58/3.59/3.60, *BnSnRK3*.69/3.70, *BnSnRK3*.71/3.72) were found located less than 200 kb. However, the identities of these gene pairs were less than 70%, indicating they were not tandem duplication events.

**Fig. 5:**
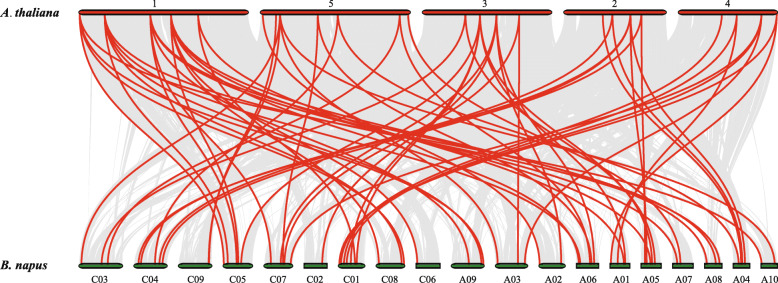
Synteny analysis of *SnRK* genes between *B.napus* and *A.thaliana*. Gray lines in the background indicate the collinear blocks within *B.napus* and *A.thaliana*, while the red lines highlight the syntenic *SnRK* gene pairs. The specie names with the prefixes ‘*A. thaliana*’ and ‘*B. napus*’ indicate *Arabidopsis thaliana* and *Brassica napus*, respectively

Futhermore, the synteny of *SnRK* gene pairs between *B.napus* genome and *A.thaliana* genome was performed. The result showed that 65 *SnRK* genes of *B.napus* exhibiting syntenic relationship with *AtSnRK* genes (Fig. 5 and Table S6), suggesting that these genes might have contributed to the evolution of *BnSnRK* gene family. To test the evolutionary constraints acting, *Ks* values, *Ka* values, *Ka/Ks* ratios and divergence time of paralogous and orthologous on *SnRK* family genes were calculated (Tables S5 and S6). The majority of segmental duplicated *BnSnRK* gene pairs had *Ka/Ks* ratios less than 1, and the mean value of *BnSnRK3* gene pairs (*Ka/Ks* = 0.30) was lower than *BnSnRK1* (*Ka/Ks* = 0.41) and *BnSnRK2* (*Ka/Ks* = 0.75). In addition, the *Ka/Ks* ratios of most orthologous *SnRK* gene pairs were less than 1, and the mean value of *SnRK2* gene pairs (*Ka/Ks* = 0.09) was much lower than that of *SnRK1* (*Ka/Ks* = 0.75) and *SnRK3* (*Ka/Ks* = 0.23). These results suggested that the *BnSnRK* gene family may have suffered robust purifying selective pressure during evolution.

### Stress-related *cis*-elements in the promoters of *BnSnRK* genes

**Fig. 6:**
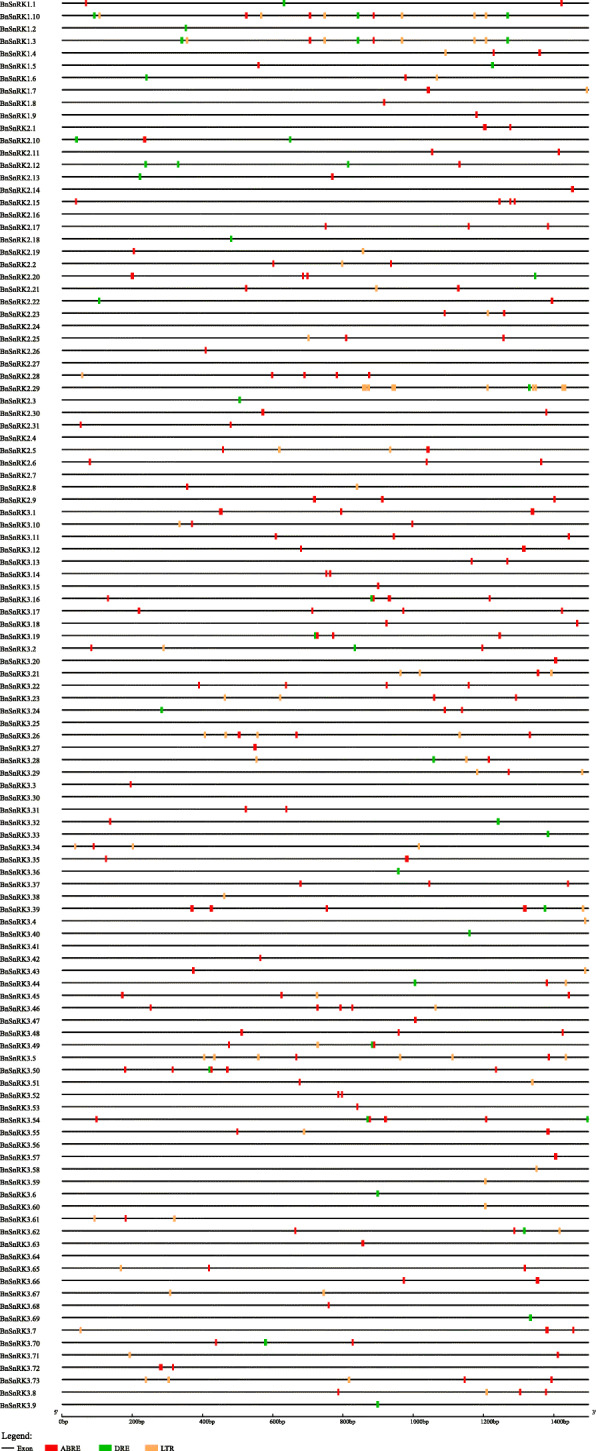
Predicted cis-elements in *BnSnRK* promoters. Promoter sequences (− 1500 bp) of 114 *BnSnRK* genes were analyzed by PlantCARE. The upstream length to the translation starting site can be inferred according to the scale at the bottom. The red, green and orange colored boxes stand for ABRE, DRE and LTR cis-elements, respectively

To understand the potential function and regulatory mechanisms of *BnSnRK* genes, *cis-*elements (1.5-kb upstream from ATG) were analyzed by using PlantCARE. Totally 104 of 114 *BnSnRK*s were identified with *cis-*elements including DRE, ABRE and LTRE, involving in dehydration responses, ABA responses and low-temperature responses (Fig. 6, Table S2). In detail, 88 *BnSnRK* genes (77.19%) had ABRE *cis*-elements, 33 *BnSnRK* genes (28.95%) carried DRE *cis*-elements, and 44 *BnSnRK* genes (38.60%) owned LTRE *cis*-elements. It was also found that most genes had more than one kind of *cis*-element types. Furthermore, the number of *cis*-elements in *BnSnRK1* (1.80) family was higher than *BnSnRK2* (1.17) and *BnSnRK3* (1.21) families (Table S2). In conclusion, the *cis-*elements analysis suggested that most *BnSnRK* genes could be responsed to diverse environmental stresses, and different subfamily genes may be regulated diversely.

### Expression profiles of *BnSnRK*s in different tissues

**Fig. 7:**
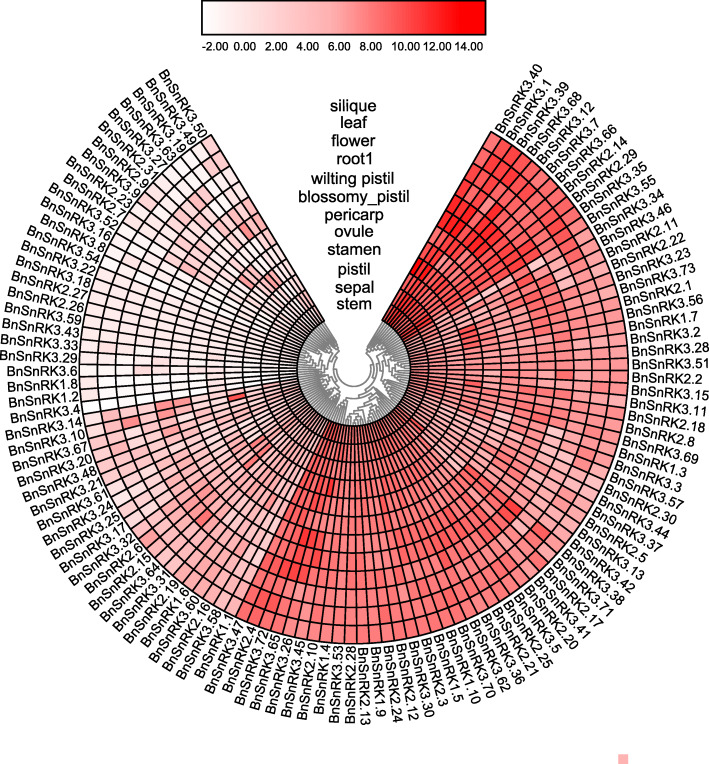
Expression profiles of the *BnSnRK* genes in different tissues. Expression data were processed with log_2_ normalization. The color scale represents relative expression levels from high (dark colored) to low (light color)

The expression profiles of 114 *BnSnRK* genes were compared among 12 tissues of the rapeseed cultivar ZS11 (Fig. 7 and Table S7). The expression pattern of *BnSnRK*s was divided into three groups. A total of 67 genes with high expression level in all examined tissues (log2-based values > 6) were classified into the group 1. For example, *BnSnRK3.40* was highly expressed in all vegetative organs with average log2-based values of 10.85. 21 *BnSnRK* genes belonging to group2 exhibited relatively low expression levels across the detected tissues (log2-based values > 2). The group3 included 26 *BnSnRK* genes, with lowest expression levels in all tissues (log2-based values < 2). Furthermore, 6 genes belonging to the group3 (*BnSnRK2*.26/2.27 and *BnSnRK*3.18/3.22/3.43/3.59) were even not expressed in all tissues. Meanwhile, the group1 contained 6 *BnSnRK1,* 21 *BnSnRK2,* 40 *BnSnRK3* genes; the group 2 had 2 *BnSnRK1,* 4 *BnSnRK2,* 15 *BnSnRK3* genes and the group 3 consisted of 2 *BnSnRK1,* 6 *BnSnRK2,* 18 *BnSnRK3* genes. These results demonstrated that *BnSnRK*s displayed diverse expression patterns*,* and genes within the same subfamily also expressed differently.

### Expression profiles of *BnSnRKs* under different abiotic stresses

**Fig. 8:**
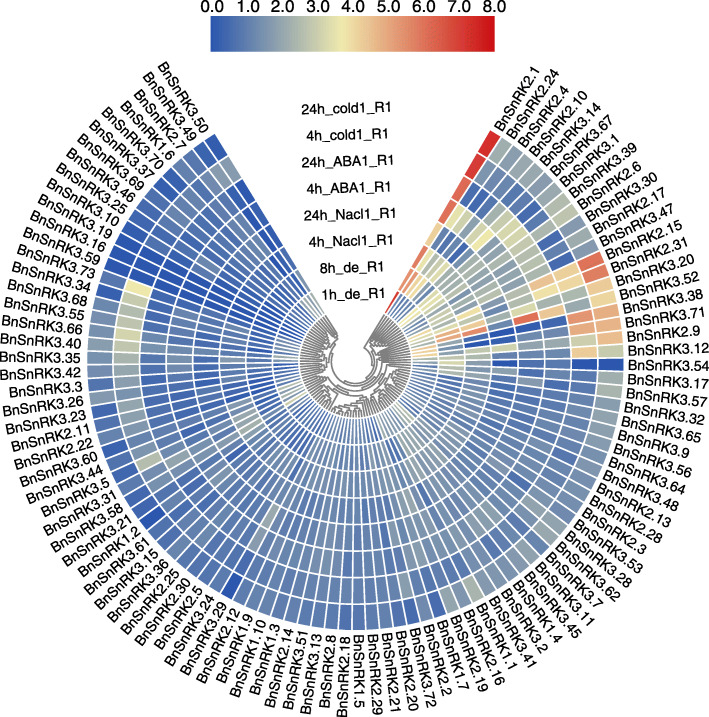
Expression profiles of the *BnSnRK* genes under different abiotic stresses. Expression data were the ratio to control values. The color scale represents expression data being processed with log_10_ normalization. The color scale represents relative expression levels from high (red colored) to low (blue color)

The expression pattern of *BnSnRK* genes under various abiotic stress were studied using the published transcriptome data of *B.napus* under drought, salinity, ABA induction and cold stresses [28]. Overall, *BnSnRK* genes significantly changed the expression level under different abiotic stresses (Fig. 8, Table S8). Multiple *BnSnRK* genes were significantly induced by diverse treatments. For example, *BnSnRK2.1* was extremely induced by all treatments. *BnSnRK3.39* incerased expression levels responding to dehydration and ABA treatments. In contrast, several *BnSnRKs* did not respond to any abiotic stresses. For instance, *BnSnRK2.12* and *BnSnRK2.13* showed almost no expression changes during all treatments. Interestingly, many genes showed opposing expression profiles under diverse treatments. For instance, *BnSnRK3.54* was highly up-regulated by dehydration, whereas was repressed by ABA and cold treatments.

### SNPs analysis of *BnSnRKs* in a core germplasm of *B.napus*

The polymorphism of *BnSnRK* genes was determined using our previous resequencing data of a core germplasm resource containing 300 accessions [29, 30]. The SNPs with MAF more than 5% in *BnSnRK* genes were selected out (Table S9). It was shown that each *BnSnRK* genes contained an average of 11.76 SNPs. In detail, each *BnSnRK1* subfamily genes included 14.00 SNPs, each *BnSnRK2* and *BnSnRK3* subfamily genes contained an average of 9.67 and 12.53 SNPs. Furthermore, the SNP density of each *BnSnRK* genes within the same subfamily was also different. For instance, there was no SNP identified in *BnSnRK1.1*, whereas there were 62 SNPs in *BnSnRK1.7*.

**Fig. 9:**
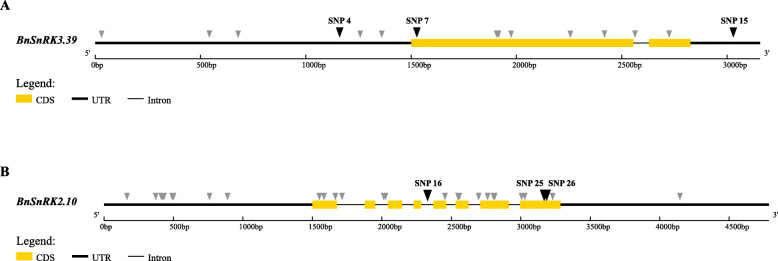
Genomic structure of *BnSnRK3.39* (**a**) and *BnSnRK2.10* (**b**). 15 SNPs and 29 SNPs were identified respectively. SNPs which significantly correlated with phenotype under unpublished data are indicated with black triangle

Moreover, a detailed SNP distribution analysis of *BnSnRK2.10* and *BnSnRK3.39* was conducted because the two genes significantly changed their expression level under drought stress (Fig. 9). It was found that there were 9 SNP loci in the 1500 bp promoter region, 19 SNPs in the exon/intron region and 1 SNP in the 3’UTR region of *BnSnRK2.10;* while we identified 6 SNP loci in promoter region, 8 SNPs in exon/intron region and 1 SNP in the 3’UTR region of *BnSnRK3.39*. Combined with phenotypic data of the core germplasm resource under drought stress (data not shown), it was found that SNP16, SNP26, SNP27 in *BnSnRK2.10*, and SNP4, SNP7, SNP15 in *BnSnRK3.39* were significantly associated with their drought tolerance.

## Discussion

In this study, 114 members of *BnSnRK* genes were identified in the *B.napus* genome, which were designated as *BnSnRK1.1* to *BnSnRK3.73* on the basis of their subfamily classification. Systematically investigation on the *BnSnRK* gene family were carried out, inculding phylogenetic relations, gene structures, protein motifs, chromosome distributions, gene duplication and *cis-*elements in the promoters. Furthermore, the deep analysis was done on the expression pattern and SNP determination of *BnSnRK* family genes using published data. This study provides a basic information for further functional characterization of *SnRK* genes to enhance plant adaptive capacity under abiotic stress.

Previous studies identified 39, 48, 44 and 34 *SnRK* genes in *Arabidopsis thaliana* [3], *Oryza sativa* [31], *Brachypodium distachyon* [32] and *Eucalyptus grandis* [33], respectively. In *B.napus* genome, *BnSnRK* gene number is much higher than diplont plants. The 114 *BnSnRK* genes were identified and classified into three subfamilies, including 10 *BnSnRK1* genes, 31 *BnSnRK2* genes and 73 *BnSnRK3* genes. More specific description showed similar member proportions of each subfamilies between *B.napus* and other species. *B.napus* originates from natural crossing of *B.rapa* (AA) and *B.oleracea* (CC) [25]. 56 and 54 *BnSnRK* genes were located in the AA subgenome and the CC subgenome, which indicated that *SnRK* genes played a similarly important functional role in both ancestral species.

Various *SnRK* subfamily genes contain different conserved domains, but all genes retained a N-terminal protein kinase domain. For example, *SnRK3* subfamily genes were reported interact with CBLs in a calcium-dependent manner because of the NAF domain permits. In addition, the NAF domain defines a set of heterologous kinases involved in diverse signaling processes, as targets of CBL calcium sensor proteins [7]. In this study, it was also found that different *BnSnRK*s subfamily genes shared the different type of conserved domains. It may suggest there is functional diversification of the *BnSnRK* gene family according to their domains.

In *AtSnRK* and *BnSnRK* gene families, different subfamily genes exhibited significant gene length and exon-intron structural divergences. In previous studies, genes with less introns were considered to have higher expression levels in plants [34, 35]. A compact gene structure with less introns allowed rapid gene activation and timely response to diverse environmental stresses [35]. However, combined with transcriptome data used in this study, we did not detect that *BnSnRK* genes with less introns showed higher expression levels than the other *BnSnRKs*.

Accumulating evidence suggested that gene activities were generally correlated with disparities in promoter regions [36]. The *cis-*elements located in the promoter regions of genes played key roles in regulating gene expression during growth and environmental changes [37, 38]. The promoter analysis revealed that *BnSnRKs* contained various types of *cis-*elements, such as DRE, ABRE and LTRE. Most of gene promoters contained at least one of these components, indicating that many of *BnSnRKs* were able to respond to diverse abiotic stresses. For instance, by combining gene expression profiles under 4 h-cold stress, the expression level of *BnSnRKs* with LTRE and ABRE *cis*-elements increased by an average of 2.71-fold, while *BnSnRKs* with no *cis*-elements only showed 1.47-fold increase. Therefore, the *cis-*elements analysis provide a clue for gene function study, especially for gene expression pattern under different stress.

Gene expression profiles provided imperative clues to map out gene functionality. Firstly, we used pre-published transcriptome data to investigate *BnSnRK* genes expression levels in diverse tissues and organs of *B.napus* [26]. The analysis results revealed that the expression patterns of these genes were divided into three groups (Fig. 7). Meanwhile, We found *BnSnRKs* in group2 contained less cis-elements in promoter than group1 and group3. Specifically, each gene in group1 contains an average of 2.84 ABRE, 0.40 DRE, 0.93 LTRE, and each gene in group3 contains 2.81 ABRE, 0.38 DRE, 0.30 LTRE, whereas each of group2 gene only has 2.00 ABRE, 0.19 DRE, 0.57 LTRE. These evidences suggested that *BnSnRKs* activities were correlated with disparities in promoter regions.

The roles function of some *BnSnRKs* reponding to diverse abiotic stresses were also derived. According to the drought stress data (Table S8), the responsive gene *BnSnRK2.10* was orthologous to *AtSnRK2.3*, which regulated ABA synthesis and signaling responding to drought in *A.thaliana* [39], indicating identical function of *BnSnRK2.10* in responding to drought stress*. BnSnRK2.24,* exhibited extremely expression changes in drought, salt stresses and ABA induce, while its orthologs *AtSnRK2.2,* could also respond to osmotic stress and ABA induction *in A.thaliana,* indicating that *BnSnRK2.24* may share similar functions in *B.napus* [40, 41]. However, *BnSnRK3.3*9 gene, which could be significantly induced under diverse stresses, was only existed in *B.napus.* Moreover, combined with resequencing data of the core germplasm resource, SNPs of each *BnSnRK* genes were identified. And the SNP distribution analysis of *BnSnRKs* such as *BnSnRK2.10* and *3.39,* could further provide functional molecular markers and alleles for these *BnSnRK* family genes.

This study provides a comprehensive knowledge of *SnRK* gene family in *B.napus*. It gives an important implication for further understanding the biological functions of individual *BnSnRK* genes in *B.napus*. However, the study only provided preliminary characterization of *BnSnRK* genes and large functional validation work need to be done in further work to understanding the roles of *BnSnRK* family.

## Conclusion

*SnRK* genes play important roles in signaling pathways including responses to biotic and abiotic stresses in plants. In this study, a comprehensive study of *SnRK* gene family in *B.napus* was performed. A total of 114 *BnSnRK* genes were characterized and divided into three subfamilies, which showed high similarity in gene structure and motif composition within the same subfamily. Phylogenetic comparison and synteny analysis of *SnRK* genes between *A.thaliana* and *B.napus* provide valuable clues for the evolutionary characteristics of the *BnSnRK* genes. Moreover, the *cis-*acting elements, gene expression and SNPs distrubution of *BnSnRK* family were also determined. These results provide an important information for further understanding biological functions of *BnSnRK* genes in *B.napus*.

## Methods

### Identification of *BnSnRK* family genes in the *B.napus* genome

The amino acid sequences of *SnRKs* gene family in *A.thaliana* were downloaded from the NCBI (https://www.ncbi.nlm.nih.gov/). The homologous genes of *AtSnRKs* in *B.napus* genome were blasted in the reference genome of rapeseed cultivar Ningyou 7 (http://ibi.zju.edu.cn/bnpedigome/download.php?con=ny7) [27]. Hidden Markov Model (HMM) and BLASTP program were applyed for preliminary identification of *BnSnRK* proteins. Local BLASTP (E-value-20) searches were performed based on Hidden Markov Model (HMM) profiles of *SnRK* gene domains from the Pfam database (http://pfam.janelia.org/). All candidate sequences of *SnRK* genes were reconfirmed through the SMART database (http://smart.embl-heidelberg.de/) [42], the NCBI Conserved Domain database [43] and the Pfam database [44]. Moreover, the number of amino acids, molecular weights (MW) and isoelectric point (pI) of each *SnRK* protein were calculated using tools from ExPASy (http://www.expasy.ch/tools/pi_tool.html).

### Phylogenetic analysis and classification of *SnRK* gene family in *B.napus*

The ClustalW with default parameters was used for multiple sequence alignment of 114 *BnSnRK* non-redundent amino acid sequences [45, 46]. Based on the alignments, a phylogenetic tree was constructed using MEGA 7.0 by the Neighbor-Joining (NJ) method [46], with the following parameters: poisson model, pairwise deletion and 1000 bootstrap replications. Unrooted NJ tree of all *SnRK* protein sequences from *A.thaliana* and *B.napus* was also constructed using MEGA 7.0.

### Identification of motif compositions and gene structures

To identify conserved motifs of *BnSnRK* proteins, the Multiple Expectation Maximization for Motif Elicitation (MEME) online program [47] (http://meme.sdsc.edu/meme/itro.html) was performed with the following parameters: number of repetition = any, maximum number of motifs = 10; and optimum motif length = 6 to 100 residues. The exon-intron structures of *BnSnRK* family genes were analyzed using the Gene Structure Display Server online program (GSDS: http://gsds.cbi.pku.edu.ch) [48].

### Chromosomal location and gene duplication

The chromosomal positions of all *BnSnRK* genes were mapped to 19 chromosomes of the rapeseed genome according to the physical location information from the NY7 genome database using MapChart version 3.0 and Circos [49, 50]. To identify gene duplication, all *B.napus* gene sequences were aligned using BLASTP, with an e-value of 1e-10. MCScanX with default values was used to classify the duplication patterns of the *SnRK* into segmental, tandem duplications [51]. The definition of tandem duplication is a chromosomal region within 200 kb containing two or more genes [52]. To exhibit synteny relationships of the orthologous *SnRK* genes between *B.napus* and *A.thaliana*, the syntenic analysis maps were constructed using python script written by ourselves. Non-synonymous (*Ka*) and synonymous (*Ks*) substitution of each duplicated *BnSnRK* gene were calculated using *KaKs*_Calculator 2.0 [53]. Divergence time was estimated using the formula T = *Ks*/2R, where R is 1.5 × 10–8 synonymous substitutions per site per year [53].

### *Cis-*elements in promoter regions of *BnSnRKs*

Upstream sequences (1500 bp) from the start codon of each *BnSnRK* gene was extracted from the genome sequence of NY7, and then used for *cis-*element distributions in promoter regions using PlantCARE software (http://bioinformatics.psb.ugent.be/webtools/plantcare/html/) [54].

### Expression patterns of *BnSnRKs*

Transcriptome data of 12 different *B.napus* tissues were obtained from a previous study by Sun et al. [26] under the project ID of PRJNA394926 in the NCBI. Moreover, transcriptome data of *B.napus* under dehydration, salt, ABA induce and cold stress condition were described in Zhang et al. [28]. The data was available at (https://bigd.big.ac.cn/) under the project ID of CRA001775. Differential expression analysis of *BnSnRKs* was performed using the DSEeq2 R package and the heatmaps were created by TBtools software.

### SNP distribution analysis of *BnSnRKs* in *B.napus* accessions

The SNPs in the coding regions of *BnSnRK* genes were extracted from the total SNPs of 300 *B.napus* accessions, which were determined by the genome re-sequencing in our previous studies [29, 30]. High-quality SNPs with MAF greater than 5% and missing rate less than 50% were used for further analysis. All high-quality SNPs were mapped to the “Darmor-*bzh*” genome (*B.napus* v4.1 genome, http://www.genoscope.cns.fr/brassicanapus/data/).

## Supplementary information

**Additional file 1: Table S1.** The information of *A. thaliana SnRKs* in queries. **Table S2.** Characteristics of *SnRK* family in *B.napus*. **Table S3.** List of the 114 *BnSnRK* genes identified in this study. **Table S4.** Conserved amino acid motifs and annotation of *BnSnRKs*. **Table S5.** Syntenic blocks and *Ka/Ks* values of *BnSnRK* genes. **Table S6.** One-to-one orthologous relationships between *B.napus* and *A.thaliana*. **Table S7.** The expression profiles (log2-based values) of the *BnSnRKs* under different tissues. **Table S8.** The expression profiles (ratio to control values) of the *BnSnRKs* under different abiotic stresses. **Table S9.** List of the *BnSnRK* SNPs identified in this study.

## Data Availability

All data analyzed during this study are included in this article and its Additional files.

## Abbreviations

ABA: Abscisic acid
ABRE: ABA responses
AMPK: AMP-activated protein kinase
*AtSnRK*: *Arabidopsis thaliana SnRK*
*BnSnRK*: *Brassica napus SnRK*
BLASTP: Basic local alignment search tool-protein
CBLs: Calcium sensor calcineurin B-like proteins
*CIPK*: CBL-interacting protein kinases
DRE: Dehydration responses element
GSDS: Gene structure display server
HMM: Hidden markov mode
LTRE: Low-temperature responses element
ROS: Reactive oxygen species
SAPK: Stress-activated protein kinase
*SnRK*: Sucrose non-fermenting 1 (SNF1)-related protein kinases
SOS: Salt overly sensitive
